# A Review of Ferdous al-Hekma fil-Tibb by Ali ibn Raban Tabari

**Published:** 2015-05-17

**Authors:** Mohammadreza Ardalan, Kazem Khodadoust, Elmira Mostafidi

**Affiliations:** 1 Kidney Research Center, Tabriz University of Medical Sciences, Tabriz, Iran;; 2 Philosophy and History of Medicine Research Center, Tabriz University of Medical Sciences, Tabriz, Iran;; 3 Kidney Research Center, and Department of Pathology, Imamreza Hospital, Tabriz University of Medical Sciences, Tabriz, Iran.

**Keywords:** *Ferdous al-Hekma*, *Paradise of Wisdom*, *Raban Tabari*, *Abbasid Caliph*

## Abstract

T Ferdous al-Hekma (Paradise of Wisdom) is one of the oldest medical texts in the Islamic world written in Arabic in 850 AD by Ali ibn Raban Tabari. He was a Persian physician who moved from Tabaristan (Mazandaran province of modern day Iran) to Samarra during the reign of the Abbasid Caliph al-Mutawakkil (847-861 AD).

We studied the book of Ferdous al-Hekma fil-Tibb, in an attempt to comprehend its general outlook on diseases of different organs, their classifications and the associated signs and symptoms.

The book is one of the earliest medical pandects of the period of translation, adaptation and expansion of knowledge in the Islamic world during the 9^th^ century AD. Tabari was mainly influenced by Hippocrates, Galen and Aristotle, as well as his contemporaries Johanna ibn Massavieh and Hunayn ibn Ishaq. The book is written in thirty chapters in a total number of 308 subtitles. In each part there is an introduction to the symptomatology, followed by organ specific diseases and therapeutic recommendations.

Symptoms and physical signs of different diseases are vividly described in Ferdous al-Hekma, and some of them are even understandable for contemporary medical students.

## Introduction

**Figure 1:**
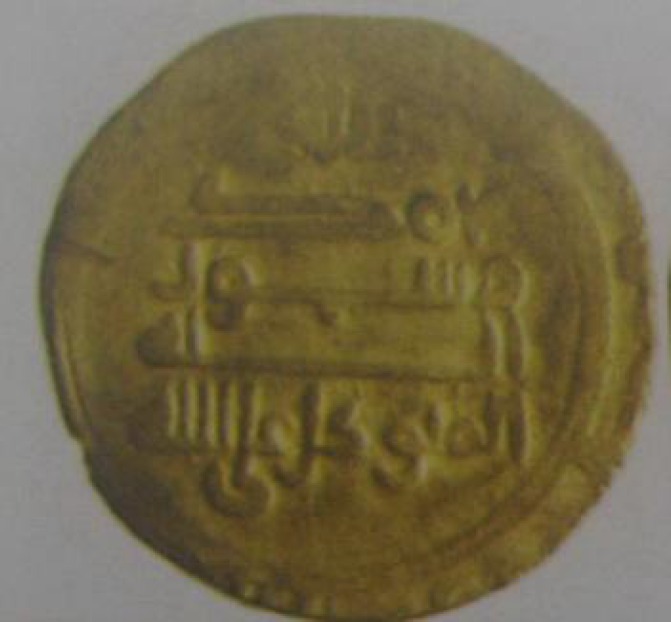
Coin of the Abbasid Caliph Al-Mutawakkil (847 AD). During his reign Ali ibn Raban Tabari moved from Tabaristan to Baghdad (Malek Museum, Tehran, Iran).

Ferdous al-Hekma (Paradise of Wisdom) is one of the oldest medical texts in the Islamic world written in 850 AD in Arabic. The writer, Ali ibn Raban Tabari (?-882 AD), was a Persian Muslim who moved from Tabaristan (the present Mazandaran province of Iran) to Samarra during the rule of the Abbasid Caliph al-Mutawakkil (847-861 AD) (1, 2) (Figure 1).

The ninth century was the beginning of the nationalistic rebellion movements of Bābak Khorramdin **(**Xorramdin Pāpak) in Azerbaijan (816-837 AD), and the great achievements of Yaqub Leis Saffar (840-879 AD) in Sistan. The Saffarids recaptured major territories of Persia from the Abbasid Caliph and appointed Naser ibn Ahmad Samani as the ruler of Balkh in Transoxiana. These movements marked the revival of Persian art, culture, science and language in the following century (2, 3).

**Figure 2:**
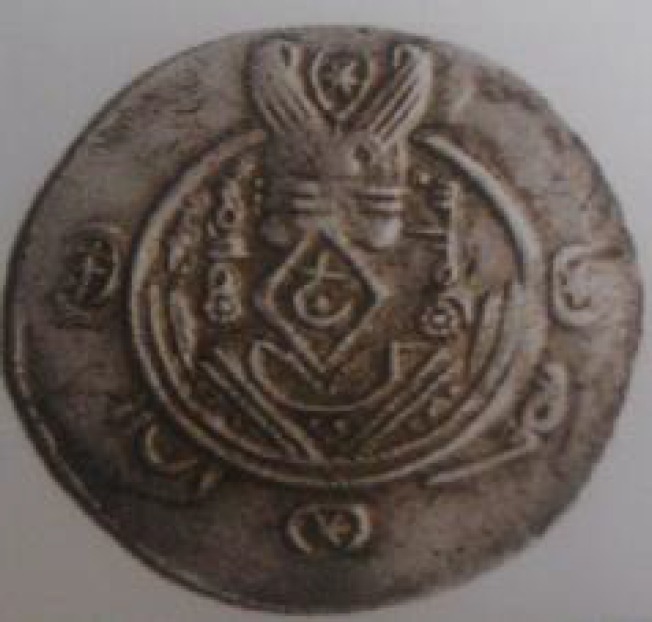
Local rulers of Tabaristan tried to continue and preserve Persian Sasanid traditions up to two centuries after Arab conquest of Persia. A coin that shows crown and wings, 775-780 AD. (Malek Museum, Tehran-Iran)

In the opening pages of his book Tabari writes about the period and being forced to migrate to the court of the Abbasid Caliph (4) (Figure 2). His words are: … “A bitter event led me to leave my motherland and a nobleman ordered me his companionship, and finally I entered the city of Samarra in the third year of al-Mutavakkil’s rule …” (4). Tabari was a master of Arabo-Greek and Persian medical knowledge and his work is the oldest technical medical book written by a Persian physician in Arabic (3).

Tabari was mainly influenced by Hippocrates and Galen, and at the beginning of his book he acknowledges Galen, Hippocrates and Aristotle and recognizes the inspirations and guidance of his contemporaries including Johanna ibn Massavieh and Hunayn ibn Ishaq. Tabari completed his book in the third year of the reign of Caliph al-Mutawakkil (probably in 850 AD). He was the teacher of Rhazes and may have died in 882 AD (4, 5).

The name of Ali ibn Raban Tabari has been mentioned by Ibn al-Nadim (died 998 AD) in his book Al-Fehrest, and he has also listed some of Tabari’s writings including: Ferdous al-Hekma, Essay on Possessions, Medicines and Herbs. In his book Tabaghat Pezeshkan (Physicians and their Ranking), Ibn Abi Usaibia has attributed two other works to Tabari, including one on health, and one on the understanding the life. In the books of Tarikh-e- Tabarestan (History of Tabaristan) and Kashf al-Zonon, the two pandects Bahr al-Favaa’ed (Ocean of Advantages) and Din Va Daolat (Power and Religion) are attributed to Tabari (3).

Ferdous al-Hekma is written in thirty chapters in a total number of 308 subtitles. The first five chapters are mainly dedicated to temperaments, humors and the general aspects of health. Then the diseases of different organs and their treatments are discussed in detail. In each part there is an introduction to symptomatology (4, 5). The importance of this book in the development of medicine was first discovered by the British orientalist Edward Granville Browne (1862-1926) (6).

With the recommendation and encouragement of E.G. Browne, in 1922 M.Z. Seddiqi collated and compared the three versions of Ferdous al-Hekma, including the British museum manuscript, the Berlin manuscript, and the Indian Gotha manuscript. In 1928 a complete copy based on the findings of Seddiqi was published in Arabic by Kaviani Publications in Berlin. Years later this version was translated into Persian by S.A. Madani and A.H. Broujerdi and published by Mehramin Publications in Tehran in 2008 (4). What follows is based on the English translation of excerpts from the above-mentioned Persian translation of Ferdous al-Hekma.

In this work we studied the Persian translation of Ferdous al-Hekma thoroughly and tried to find the passages on symptoms, signs and physical examinations associated with different disorders. We tried to comprehend the content of those passages and compared them against modern medicine. At the same time we studied the main historical sources on early Islamic periods to get a more vivid outlook on the period.

***Passages***

**A General Outlook on Clinical Medicine**

In the opening pages of his book Tabari writes about his main objectives and outlooks as a thinker and physician, and offers valuable ethical messages. “It is my wish that anyone who reads this book, thinks about it deeply and kindly corrects my flaws, and I shall be indebted to such persons… (5). [Medicine] is the science of protecting health and preventing pain and suffering, and this is unattainable unless science and practice are joined… (4). This practice [medicine] is called Hekmat, which is derived from divinity. Therefore no one deserves to experience such nobility unless he is adorned with generosity, kindness and devotion and is not driven by greed. A physician should be compassionate toward his patients… (5). He should alert his colleagues to their flaws, and in doing so, he will gain a high standing among them… (4). A physician should prescribe medicine after physical examinations and should never be arrogant and take excessive pride in his skills. As Hippocrates asserts, “Life is short and knowledge is long, opportunities are fleeting, time is passing, experiences are perilous and destiny is strict…” (4).

**Signs of internal diseases**

There are seven signs of internal diseases: 1) those that appear on the face. Yellow complexion, pale lips and pedal edema denote liver coldness, darkness of the face and blanched lips indicate spleen edema, and redness of the face along with a high fever indicate lung inflammation; 2) signs that can be found on extremities. For example pain in the right clavicle indicates a liver problem; 3) signs that should be sought by palpation. For instance a round area of inflammation in the lower ribs area denotes liver inflammation and if the pain area is rectangular, it indicates inflammation of the liver capsule; 4) decreased appetite; 5) [the ability of] excretion along with coughing is a sign of lung infection; 6) discomfort in different organs; and 7) signs that can be found by asking the patient about his disease…. As Hippocrates says, “The disease by itself guides us…” (4).

In this passage there is an interesting distinction between disease signs and symptoms, and a vivid description of the different stages of physical examination including inspection and palpation. Tabari also mentions some important general and organ-specific signs that can be used to diagnose diseases of different organs. The section on the referral pain of the liver in the clavicle is very interesting and can be found in modern books of medicine under the topic of physical examination.

**Signs of paralysis and convulsion**

If all extremities are paralyzed and only the face is spared, the disease pertains to the vertebral column, but if all parts of the body are involved, the brain is damaged…. If there is hemifacial paralysis, there will be mobility on the normal side and the intact muscles will retract the affected side toward themselves… (4).

There is a relatively accurate description of facial paralysis and the consideration that the more deviated side of the face actually is the normal side. It is interesting that this condition was well known to a Persian physician in the 9^th^ century, but has been named after the Scottish anatomist Charles Bells (1774-1842) (7).

**Signs of intestinal disease and diarrhea**

If the pain is in the upper umbilical area, the small intestine is involved… and if the pain is in the sub-umbilical area, the lower (large) intestine is involved. If the pain is intermittent, the upper (small) or lower intestines are involved… (4).

The distinction between large and small intestinal pathology is difficult and even modern textbooks on physical examination consider the abdominal surface distribution of pain as a guide to find the source of pain. For example duodenal pain often happens in subxiphoid areas. involvement of the third part of the duodenum, jejunum and also proximal ileum pain often radiate around the umbilicus (8).

**On dyspnea and asthma**

Respiratory organs include the lung, larynx and a partition that exists between the chest and the abdomen and is called the diaphragm… and in pleuritis the pain radiates to the neck or the brain and disturbs consciousness…. (4). 

In the paragraph above, the anatomical considerations about the radiating pattern of pain are interesting as these symptoms are used in contemporary medicine as well. 

**Signs of stomach disease and ulcers**

If the pain appears on the interscapular areas of the back, it may have originated from the esophagus because the stomach is adjacent to the lumbar area. If we feel such pain while eating spicy food, it is a sign of stomach inlet ulcer, and if the painful area is lower, the ulcer is located inside the stomach, and if the pain originates in the duodenum, it is felt within te abdomen…(4) 

In the above-mentioned passages, it is noticeable that reflux esophagitis and esophageal spasm were considered a cause of chest pain that can mimic angina. Peptic ulcer pain could radiate to the back and the upper left quadrant. Hoarseness and recurrent laryngitis could also be a sign of gastroesophageal reflux (8).

**Signs of liver disease**

The patient is thirsty, his mouth is dry, appetite is decreased, urine is [deep yellow], and his pulse is rapid… if inflammation appears in the upper surface of the liver, the patient feels pain on the right clavicle [particularly] during respiration. Pain on the right side and coughing happens because the congestion is adjacent to the lung and the diaphragm…. If the inflammation develops on the lower part of the liver, it is less painful than congestion in the upper part... if the congestion happens on the lateral surface of the liver, it is similar to the first type… (4). 

Liver pain can be hard to identify and localize because pain from abdominal organs, such as the liver, is often experienced as a vague pain in the right hypochondrium and epigastrium, which may lead one to suspect liver abscess or cancer. Patients with liver disease may report more abdominal pain than other individuals, and the pain is worse after meals. Biliary colic and acute cholecystitis must also be considered in patients with liver disease because the prevalence of gallstones is increased in patients with cirrhosis. Pyogenic liver abscess could present with fever and upper right quadrant pain, while sub diaphragmatic abscess could present with dyspnea, chest pain and even hemoptysis.

Irritation of the central part of the diaphragm radiates to the shoulder. The combination of lower chest pain and ipsilateral shoulder pain is highly suggestive of diaphragmatic pleural disease. Mediastinal pain is less severe and is located in midline and radiates to the back in the interscapular area. It is very interesting that this pattern of referral pain has been described in modern textbooks as well (9, 10).

**Signs of hemorrhage in upper and lower organs**

Blood that is vomited originates from the stomach, and blood that comes from the lung or pharynx is clotted. Blood from the lung is dark and is associated with pain and coughing… and is mixed with puss… and is more diluted and foamy… (4)

In the above-mentioned passages there is an interesting distinction between hematemesis and hemoptysis. The latter can be caused by a wide range of disorders, but massive hemoptysis is most frequently caused by tuberculosis, bronchiectasis, lung abscess, cancer, and Aspergilloma. These conditions were probably common in those periods (10, 11).

**Signs of pain in the large intestine**

If someone has large intestine problems…, he feels the pain in his kidney. Large intestine pain happens in the lateral part of the abdomen and is expandable… but kidney pain is constant and remains confined to one area, does not spread and is [often] located above the waist… And if [the patient] lies in the prone position, he feels heaviness in the kidney area…. In patients who complain from flatulence and intestinal air entrapment, if you palpate the affected area, you can feel a lump…. If air entrapment is the cause of pain, it radiates from one part to the other part…. In constipation the patient feels severe pain, pressure and a tearing sensation … (4).

The passage above contains some very interesting information. Flatulence and air entrapment within the intestinal loop are sometimes mistakenly diagnosed as kidney pain, a fact that has been pointed out in modern physical examination books (12).

Pain that originates from the right colon and transverse colon pain radiate toward the sub-umbilical region, as is the case in ulcerative colitis. In partial obstruction of recto-sigmoid colon the pain is distributed in the lower left abdominal quadrant (8).

**Types of pulses in different diseases:**

…in pleural infection the pulse is rapid and full, although often it is not completely full, but it is rapid. If the pulse rate is increased it means that the lung is involved… sometimes the patients’ pulse converts to serrated pulse that is not regular and changes, and it is similar to the teeth of a saw… the pulse of persons with lung inflation and infection is small and rapid and moves like a wave… (4).

Tachycardia is a sign of fever, but decreased intravascular volume, hypoxia and pain are some other causes of tachycardia in a patient with cardio-thoracic disease. It could also be an early sign of pleural effusion, pericarditis and myocardial involvements (7). 

## Discussion

In a review of the passages above, we found that Ferdous al-Hekma is full of considerations about the physical signs and symptomatology of diseases. Ferdous al-Hekma is one of the first compendiums that introduced Greco-Roman medicine to the Islamic world. During the Greek period and the medieval times, diseases were considered to be the result of an imbalance in the four humors (6), and the concept of anatomy-based nosology of the disease did not yet exist. Hippocrates (370-460 BC) developed the principles of clinical observation and interpretation of patients’ complaints. The next great Greek physician lived 500 years after Hippocrates and was Galen of Pergamum (129-216 AD). His ideas influenced medical thinking for the next 1500 years and were accepted as the ultimate truth until Vesalius (7). The idea of anatomical thinking took many years to develop, and was constantly suffocated by the overfull legacy of humors pathology. Vesalius and his work the Humani Corporis Fabrica Libri Septem were a new start. In this work, Vesalius described his own observations rather than justify his anatomical findings through Galen’s theories. His works were promoted by Giovanni Battista Morgagni in the 18^th^ century. Such revolutionary thinking established that diseases are not the product of unbalanced humors, but rather originate from organs. Viennese physician Leopold Auenbrugger (1722-1809) started to use the percussion of the chest as a diagnostic tool, an innovation that was later revived by Jean Nicolas Corvisart. Physicians have always tried to examine the diseased organ by using their senses, and with those ideas Joseph Skoda (1805-1881) invented auscultation. A strong connection between physical diagnosis and autopsy later created a great achievement in physical diagnosis (9). 

It should be noted that some of the physical signs discussed by Tabari in Ferdous al-Hekma are described so vividly that they are understandable by contemporary medical students (10, 11).

Ferdous al-Hekma is a junction between the early translation movement and the adaptation and expansion of medical knowledge in the Islamic world. Tabari’s very close predecessor was Hunayn ibn Ishaq (800-877 AD), a great Nestorian physician and translator of Galen’s works from Greek to Arabic and Syriac in the early 9^th^ century. He dedicated his first work, which was the translation of a part of Galen’s book on dissection in 829 AD, to Gabriel ibn Bakhtisho (died 829 AD), the great physician of Bait-al-Hekma and physician of Caliph al-Ma’mun (813-833 AD). Another important influence on Tabari was Johanna ibn Massavieh, who was also a Persian Christian physician and Gabriel ibn Bakhtisho’s student. He was a renowned physician in the Islamic world during the 9^th^ century and died in 857 AD in Samarra (12).

Greco-Roman medicine was preserved and expanded in the Arabic language as it was the lingua franca of the Islamic world during the Dark Ages (500 to 1050) and early Middle Ages. In these centuries Europe entered a period of intellectual darkness while the Islamic world was a sanctuary for scientific ideas to flourish. Ferdous al-Hekma created a new arena for other great medical compendiums to appear in the Islamic world (11, 13). In Ferdous al-Hekma the considerations on physical signs are concise, direct and practical. The chapters devoted to examination of pulse and urine and dental medicine, however, are not as detailed as they are in Hidayat al-Mutaallimin fi al-Tibb and Al-Hawi by Rhazes (14-16). The major novelty of Ferdous al-Hekma is the considerations about the signs and symptoms of diseases, as is obvious in the passages that have been presented in this article. 

In conclusion, Ferdous al-Hekma fil-Tibb (Paradise of Wisdom in Medicine) by Ali ibn Raban Tabari shows the strength and depth of medical knowledge in the early Islamic period. Greco-Roman medical heritage was introduced to the Islamic world and then reentered Europe during the middle Ages, and the achievements in Islamic medicine greatly influenced the modern scientific movements in the following years.
